# Change isn't always better

**DOI:** 10.7554/eLife.10054

**Published:** 2015-08-14

**Authors:** Heather Schofield, Marina Pasca di Magliano

**Affiliations:** Program in Cell and Molecular Biology and the Medical Scientist Training Program, University of Michigan, Ann Arbor, United States; Program in Cell and Molecular Biology, Department of Surgery and Department of Cell and Developmental Biology, University of Michigan, Ann Arbor, United Statesmarinapa@umich.edu

**Keywords:** pancreatic cancer, differentiation, pancreatitis, human, mouse

## Abstract

Maintaining the identity of acinar cells in the pancreas could help to prevent the development of pancreatic cancer.

**Related research article** Krah NM, De La O JP, Swift GH, Hoang CQ, Willet SG, Chen Pan F, Cash GM, Bronner MP, Wright CV, MacDonald RJ, Murtaugh LC. 2015. The acinar differentiation determinant PTF1A inhibits initiation of pancreatic ductal adenocarcinoma. *eLife* **4**:e07125. doi: 10.7554/eLife.07125

**Image** Pre-cancerous pancreatic lesions develop from acinar cells that have undergone a transformation to a duct-like state (green)

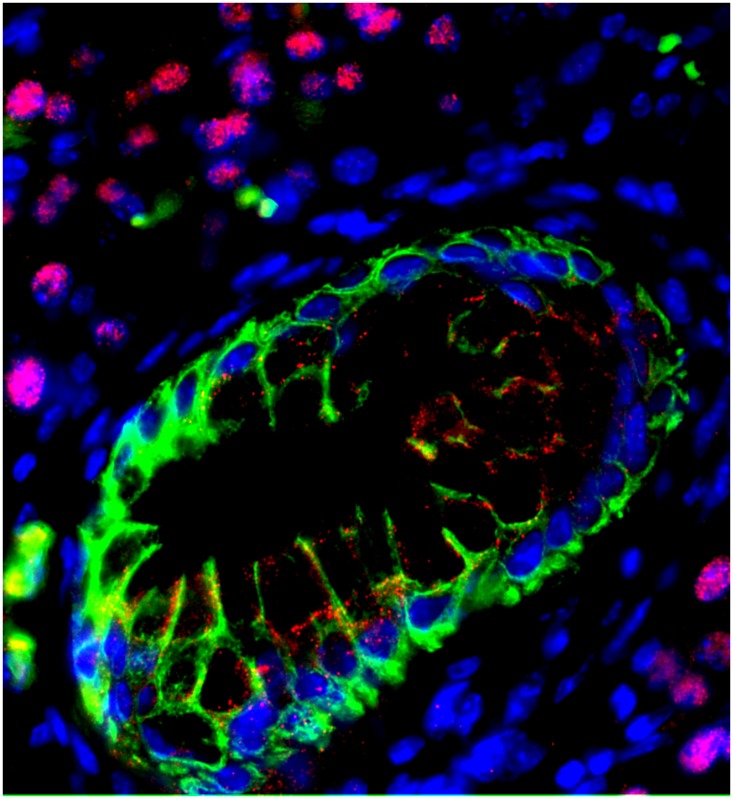

Pancreatic cancer is the fourth most common cause of cancer-related death in the United States, with only 6% of people surviving for five years after diagnosis. Now, in eLife, Charles Murtaugh and co-workers – including Nathan Krah as first author – shed new light on the process that leads normal pancreatic cells to become cancer cells (Krah et al., 2015).

The exocrine pancreas is the part of the pancreas that produces the enzymes that help to digest food, and is also the region where pancreatic cancer arises. It remains unclear how normal exocrine cells – two types of which are the acinar and ductal cells – become cancerous. Pancreatic cancer – as well as the pre-cancerous lesions that precede it – consists of cells that resemble ductal cells. It had thus been assumed that ductal cells are the origin of this disease. However, this notion has been challenged by recent experimental evidence from genetically engineered mice that suggests that acinar cells are in fact the most common source of pancreatic cancer (for review see Puri et al., 2015).

For acinar cells to give rise to cancer, they first must lose their acinar characteristics and become duct-like, in a process known as acinar-to-ductal metaplasia (or ADM). Then, these duct-like cells transform into cancer precursor cells and, in time, become cancer cells. This cancerous transformation is associated with the presence of a form of the *Kras* gene that causes cells to divide more often (known as the *Kras* oncogene). Experimentally, the transformation of the duct-like cells into a cancerous form is modelled in mice that are engineered to express the *Kras* oncogene. These mice develop small pre-cancerous lesions in their pancreas that only arise from a small subset of the acinar cells, despite mutant Kras expression in all cells.

Krah et al. – who are based at the University of Utah, Vanderbilt Medical Center and University of Texas Southwestern Medical Center – sought to determine what normally protects against the *Kras*-mediated transformation in acinar cells; that is to say, the safety mechanisms that are bypassed during the formation of pre-cancerous lesions.

Since the first step of pancreatic cancer development is the ADM process that causes the acinar cells to become duct-like, Krah et al. hypothesized that preventing ADM from occurring may protect against *Kras*-mediated transformation. They reasoned that PTF1A, a protein that regulates the differentiation of acinar cells from precursor cells during pancreas development is similarly required to maintain the identity of the acinar cells in the adult pancreas. Thus, PTF1A could protect against the *Kras*-mediated transformation of the cells to a pre-cancerous cell type by preventing ADM. This hypothesis was based on previous studies of other molecules that regulate differentiation and guide the development of acinar cells, because these so-called “acinar differentiation factors” have been seen to protect against *Kras*-mediated transformation. Furthermore, inactivating these differentiation factors promotes the ADM process (Shi et al., 2009; von Figura et al., 2014).

To investigate the role of PTF1A, Krah et al. first examined when and where this protein was expressed throughout the pancreas. This revealed that PTF1A is not produced in the pre-cancerous lesions of either humans or mice. However, PTF1A was expressed in adjacent acinar cells that appeared normal, suggesting that PTF1A may protect against *Kras*-mediated transformation in these cells.

Next, Krah et al. used genetic mouse models to prevent acinar cells from producing PTF1A, and observed spontaneous ADM in these animals. Expressing the *Kras* oncogene in these mice caused precursor lesions to form more rapidly and over a wider area of the pancreas than those seen in mice whose acinar cells expressed both PTF1A and oncogenic *Kras*. Together, these data show that acinar cells must lose PTF1A before they can undergo ADM, and thus become susceptible to *Kras*-induced transformation.

Furthermore, by gene expression analysis, Krah et al. showed that PTF1A controls multiple genes and pathways associated with both the initiation of pancreatic cancer and their continuous need for oncogenic *Kras* expression.

Overall, Krah et al.'s data show that acinar cell differentiation, which is maintained by PTF1A, protects against the expression of oncogenic *Kras* and helps to prevent the cells from becoming cancerous. The next open question is whether forced expression of PTF1A in the pancreas protects against *Kras*-induced transformation; this has already been shown to be the case for a different acinar cell factor called Mist1 (Direnzo et al., 2012). Further open questions include what it is that causes the accelerated rate at which ADM progresses to precursor lesions in mice that express oncogenic *Kras* and lack PTF1A (compared with mice that do express PTF1A). Since PTF1A is normally lost during the process of ADM, the absence of this protein cannot explain the accelerated rate of cancer progression. It is possible that in mice that carry oncogenic *Kras* and still retain intact PTF1A, an equilibrium between acinar cells and ADM cells occurs, whereby ADM cells can ‘choose’ to re-express PTF1A and return to an acinar fate, or progress to form pre-cancerous lesions (Figure 1). This equilibrium would be lost in mice lacking PTF1A, where cells that have undergone ADM have no choice other than to progress towards becoming cancerous. Furthermore, the effect PTF1A re-expression has in pre-cancerous lesions as well as in advanced disease merits further exploration.

**Figure 1.**
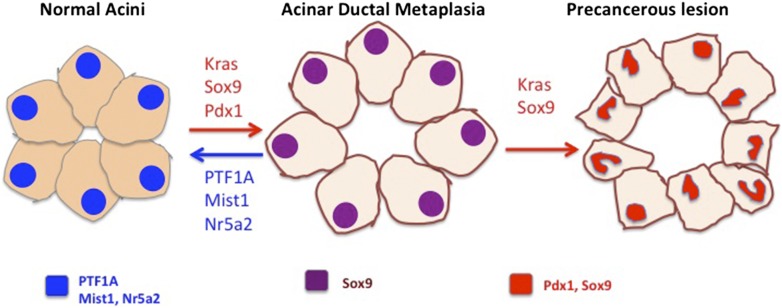
Preventing acinar cells differentiating to duct-like cells may help to prevent the development of pancreatic cancer. In the figure, the cell nuclei are colour-coded to indicate which genes are expressed. Acinar cells in the pancreas (left) express the differentiation factors PTF1A, Mist1 and Nr5a2 (blue). Krah et al. show that loss of PTF1A causes acinar de-differentiation in a process known as acinar-to-ductal metaplasia (ADM; centre). In the presence of oncogenic *Kras*, ADM leads to the formation of precancerous lesions (right) and, in time, cancer. Other factors have also been shown to be important in regulating the balance between cell differentiation and cancer: Mist1 and Nr5a2, expressed in normal acinar cells, protect from transformation. Conversely, factors that control ductal fate, including Sox9 and Pdx1, can push acinar cells toward a ductal state and accelerate the rate at which cancerous cells develop (red arrows; Kopp et al., 2012; Miyatsuka et al., 2006).

In summary, this study provides new insight into the process that triggers the development of pancreatic cancer. In doing so, it opens many avenues of exploration that will provide more insight into the biology of this disease – an essential first step towards developing new therapies.
